# A Generalized Topological Entropy for Analyzing the Complexity of DNA Sequences

**DOI:** 10.1371/journal.pone.0088519

**Published:** 2014-02-12

**Authors:** Shuilin Jin, Renjie Tan, Qinghua Jiang, Li Xu, Jiajie Peng, Yong Wang, Yadong Wang

**Affiliations:** 1 Department of Mathematics, Harbin Institute of Technology, Harbin, Heilongjiang, China; 2 School of Computer Science and Technology, Harbin Institute of Technology, Harbin, Heilongjiang, China; 3 School of Life Science and Technology, Harbin Institute of Technology, Harbin, Heilongjiang, China; 4 College of Computer Science and Technology, Harbin Engineering University, Harbin, Heilongjiang, China; Indiana University Bloomington, United States of America

## Abstract

Topological entropy is one of the most difficult entropies to be used to analyze the DNA sequences, due to the finite sample and high-dimensionality problems. In order to overcome these problems, a generalized topological entropy is introduced. The relationship between the topological entropy and the generalized topological entropy is compared, which shows the topological entropy is a special case of the generalized entropy. As an application the generalized topological entropy in introns, exons and promoter regions was computed, respectively. The results indicate that the entropy of introns is higher than that of exons, and the entropy of the exons is higher than that of the promoter regions for each chromosome, which suggest that DNA sequence of the promoter regions is more regular than the exons and introns.

## Introduction

The first concept of entropy was introduced by Shannon[8] as a measure of the complexity of a set of symbols, which can be formulated in mathematical form as:
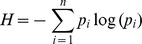
where 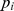 is the probability of the 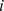-th symbol. Since then the notions of entropy appeared in many forms, such as metric entropy, topological entropy, Kolmogorov-Sinai entropy and Rènyi [7] entropy. All of the concepts were focused on one purpose: the “quantitative” description of the complexity or simplicity of a set of symbol dynamics.

The complexity of DNA sequences, as a special kind of symbol dynamics which is composed of *A,C,G,T*, can be measured by the entropy. Kirillova [5] computed DNA sequences of different organisms by the topological and metric entropies. Vinga and Almeida [9] introduced Rènyi's quadratic entropy to evaluate the randomness of DNA sequences. Zhao F, Yang H and Wang B [10] investigated the complexity of human promoter sequences by a diffusion entropy. Bose and Chouhan [3] studied the superinformation of the DNA sequence. Recently, Koslicki [6] introduced a topological entropy for finite sequences and showed the complexity of introns is higher than that of exons for each chromosome.

In this paper, a generalized topological entropy is introduced. At the same time, the relationship between the topological entropy and the generalized topological entropy is compared, which shows the topological entropy is a special case of the generalized entropy. The use of generalized topological entropy removes high-dimensional problems. This definition can get the complexity of sequences of different length. At last, we apply the generalized topological entropy to human genome to compute the complexity of introns, exons and promoter regions.

## Methods

Let 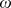 be a sequence of DNA with length 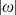, 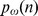 be the number of different 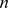-length subwords that appear in 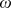. If the sequence is infinite, then the topological entropy is defined as:

### 

#### Definition 1

For an infinite sequence 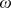 formed over *A,C,G,T*, the topological entropy is



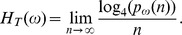
 Take a symbol sequence 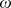 = *CGCGCGCG···* as an example. It is easily seen, for any 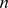, the different sequence with length 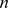 is 2, so the topological entropy of the DNA sequence *CGCGCGCG···* is:
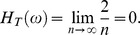



However, the length of DNA sequence is finite, by Definition 1, the complexity is zero as 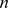 tends to infinity. Colosimo and Luca [4] showed the precise description of the shape of the complexity function, and then Koslicki defined an approximation of topological entropy 
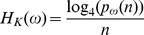
.

**Figure 1:**
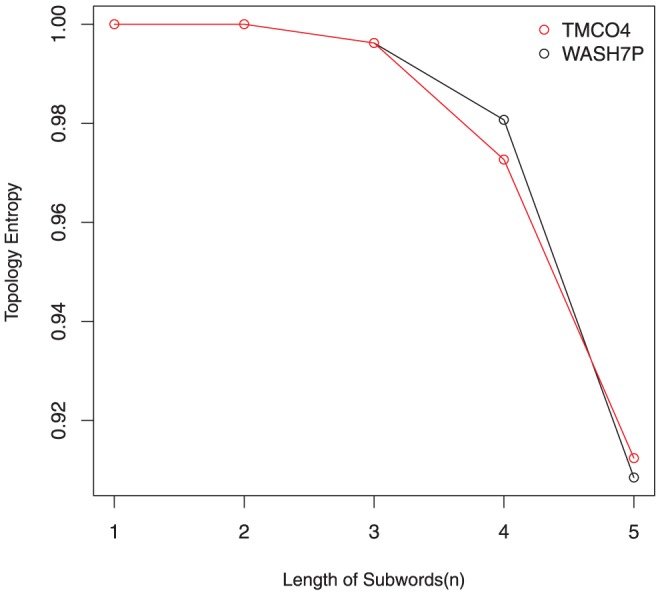
The topological entropy of the promoter regions before gene WASH7P and TMCO4. Notice that the unique number which satisfies 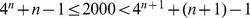 is 5. The topological entropy of promoter regions before gene WASH7P and TMCO4 are 0.908480839 and 0.912412131.

The spirit of Koslicki's method is to find a unique 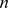, which satisfies 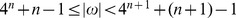, and then the complexity is decided by the subwords of length 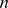. However, by comparing the topological entropies of DNA sequences, we find the complexities of subwords, which are shorter than 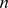, are also important. The following Figure 1 is the topological entropy of the promoter regions (2000 bp upstream before the transcription start site) before gene WASH7P and TMCO4 on chromosome 1.

Following Koslicki's definition, the complexity of the promoter region before TMCO4 is higher than WASH7P. However, considering the complexity of subwords, the fact is not the case. As a matter of fact, **the complexities of subwords all contribute to the complexity of the sequence**. Based on the idea of overall consideration, we give the following definition.

#### Definition 2

Let 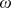 be an infinite sequence formed over *A,C,G,T*, the generalized topological entropy is 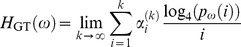
where
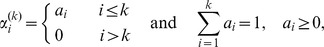
satisfies for any 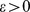, for any 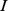, there exists 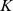, such that for all 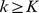, 
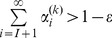
.

For infinite sequences formed over *A,C,G,T*, then 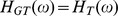. (Appendix S1)

By considering the complexity of finite sequence, we give the following generalized topological entropy.

#### Definition 3

Let 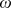 be a finite sequence of length 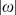. Let 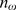 be the unique integer such that 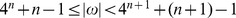.

Then for 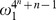 the first 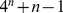 letters of 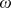 and 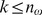,
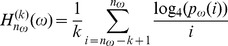



Notice that the generalized topological entropy by Definition 3 satisfies the following four important properties mentioned by Koslicki [6].

(1) 
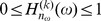
;

(2) 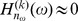 if and only if 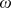 is highly repetitive;

(3) 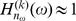 if and only if 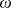 is highly complex;

(4) For different length sequences 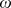,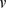 and 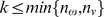, 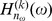
and 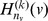 can be comparable.

It is easily seen for infinite sequence, 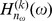 is a special case of 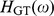. Moreover, the topological entropy defined by Koslicki satisfies 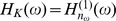.

## Applications to Human Genome

### Data

We retrieved the hg19 human genome assembly from the UCSC database and utilized Galaxy(Blankenberg [1]–[2]) to extract the nucleotide sequences of the introns, exons and promoter regions of each chromosome. The sequences that are too short would lead to significant noise. For example, the UCSC database contains exons that are only one base pair long and it is trivial to measure the complexity of such sequences. We selected randomly 100 different promoter sequences (2000 bp upstream before the transcription start site) from each chromosome, and repeated this procedure for 100 times and computed the average complexity of promoter sequences.

### Results and discussions

We used the generalized topological entropy to compute the complexity of introns, exons and promoter regions of the human genome by non-overlapping windows algorithm by Koslicki.

**Figure 2:**
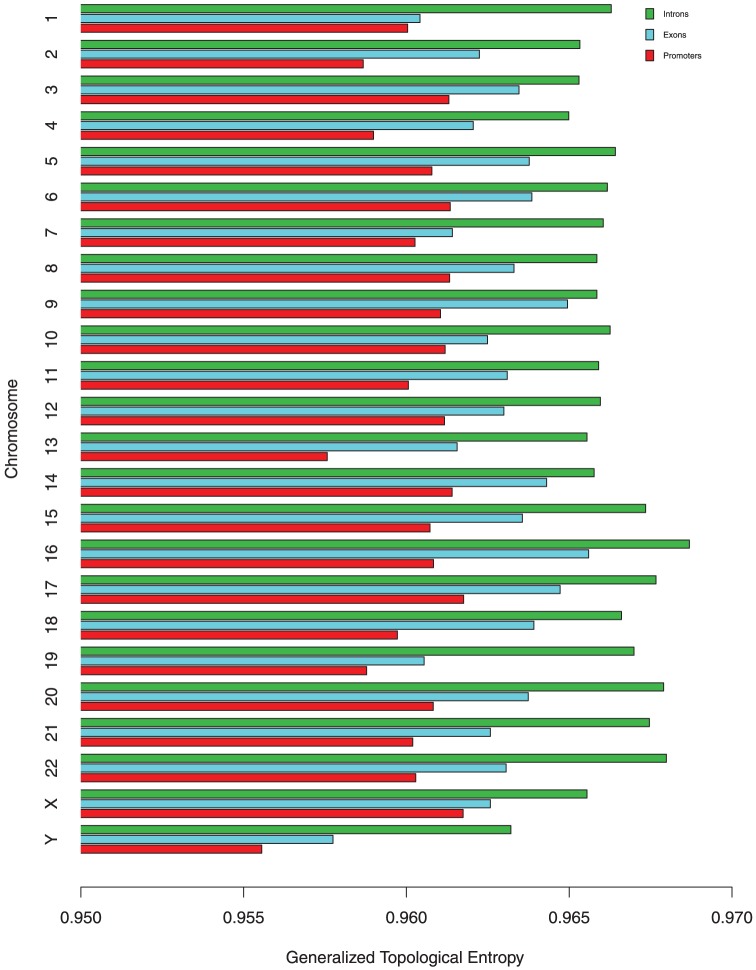
Generalized topological entropies of introns, exons and promoter regions.

Figure 2 displays the complexity of human introns, exons and promoter regions by 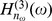. Here we took the mean of the generalized topological entropies of 100 different introns and exons sequences from each chromosome. We did ANOVA of the complexity differences among introns, exons and promoters on each chromosome and found the differences are statistical significant. (p-value<0.005, Appendix S2)

It is seen by Figure 2, the generalized topological entropy of introns is higher than exons for each chromosome, which demonstrates the structure of introns is more complex than exons. This is reasonable due to the fact the introns of DNA sequences are free from selective pressure and so evolve more randomly than exons.

Note that promoter regions are among the most conserved elements in Eukaryotic genomics, which consist of the TATA box, CAAT box, GC-enriched region and so on. Thus one would expect that the generalized topological entropy of these regions would be very low. As shown in figure 2, the mean of the generalized topological entropies of 100 different promoter regions is lower in comparison to the mean of the generalized topological entropies of 100 different introns and exons for each chromosome, which suggests the generalized topological entropy can be used to detect functional regions and regions under selective constraint.

## Conclusions

The generalized topological entropy has two advanced features. Theoretically, the definition of generalized topological entropy is a complete form of topological entropy. Practically, the use of the generalized topological entropy allows comprehensive analysis of the complexity of DNA sequences, which counts for almost all the complexities of the subwords. Besides measuring the complexity or simplicity of sequences, the generalized topological entropy can be used to detect functional regions and regions under selective constraint.

## Supporting Information

Appendix S1 **Proof of the generalized topological entropy.**
(DOC)

Appendix S2 **Complexity differences among introns, exons and promoters.**
(DOC)
