# Supplementary material for: A Generalized Topological Entropy for Analyzing the Complexity of DNA Sequences
Source: PLoS One. 2014 Feb 12;9(2):e88519. doi: 10.1371/journal.pone.0088519 (PMC3922877; doi:10.1371/journal.pone.0088519)
Supplement: Appendix S1 — Proof of the generalized topological entropy. (DOC) [file pone.0088519.s001.doc]

Appendix S1.

**Proof of the generalized topological entropy**

Notice that for any $\varepsilon>0$, for any $I$, there exists $K$, such that for all $k\geq K$, $\sum_{i=I+1}^{\infty} \alpha_{i}^{(k)}>1-\varepsilon$ is equivalent to $\sum_{i=1}^{I} \alpha_{i}^{(k)}\leq\varepsilon$ under the condition $\sum_{i=1}^{k} \alpha_{i}^{(k)}=1$.

Let

then $a\leq1$, it suffices to prove $H_{GT}(\omega)=a.$

For any $\varepsilon>0$, there exists $N$, such that $n>N$,

$|\frac{\log_{4}(p_{\omega}(n))}{n}-a|<\varepsilon$,

for the $\varepsilon, N$ above, there is $K\geq N$, such that $k>K$, $\sum_{i=1}^{N} \alpha_{i}^{(k)}<\varepsilon$, then

$$\begin{matrix} |\sum_{i=1}^{k} \alpha_{i}^{(k)}\frac{\log_{4}(p_{\omega}(i))}{i}-a|=|\sum_{i=1}^{k} \alpha_{i}^{(k)}(\frac{\log_{4}(p_{\omega}(i))}{i}-a)|\leq\\ |\sum_{i=1}^{N} \alpha_{i}^{(k)}(\frac{\log_{4}(p_{\omega}(i))}{i}-a)|+|\sum_{i=N+1}^{k} \alpha_{i}^{(k)}(\frac{\log_{4}(p_{\omega}(i))}{i}-a)|\leq2\varepsilon+\varepsilon\end{matrix}$$

which completes the proof.
