# Supplementary figures and images for: A Generalized Topological Entropy for Analyzing the Complexity of DNA Sequences
Source: PLoS One. 2014 Feb 12;9(2):e88519. doi: 10.1371/journal.pone.0088519 (PMC3922877; doi:10.1371/journal.pone.0088519)

Appendix S2.

**Complexity differences among introns, exons and promoters**


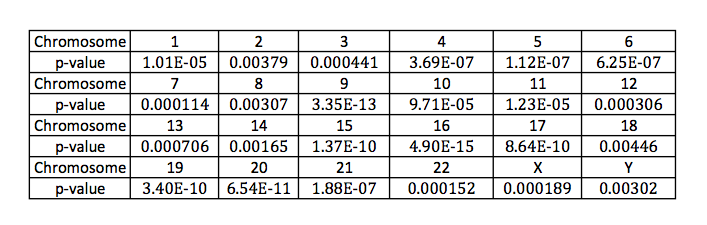

Supplement: Appendix S2 — Complexity differences among introns, exons and promoters. (DOC) [file pone.0088519.s002.doc]
